# RNA-seq data reveals a coordinated regulation mechanism of multigenes involved in the high accumulation of palmitoleic acid and oil in sea buckthorn berry pulp

**DOI:** 10.1186/s12870-019-1815-x

**Published:** 2019-05-20

**Authors:** Jian Ding, Chengjiang Ruan, Wei Du, Ying Guan

**Affiliations:** 1 Key Laboratory of Biotechnology and Bioresources Utilization, Ministry of Education, Institute of Plant Resources, Dalian Minzu University, 18 Liaohe West Road, Dalian, 116600 Liaoning China; 2 Institute of Berries, Heilongjiang Academy of Agricultural Sciences, 5 Fansheng Street, Suiling, Heilongjiang, 152230 China

**Keywords:** Palmitoleic acid, *Hippophae* L., Fatty acid biosynthesis, Oil accumulation, Berry pulp oil, Non-seed tissue

## Abstract

**Background:**

Sea buckthorn is a woody oil crop in which palmitoleic acid (C16:1n7, an omega-7 fatty acid (FA)) contributes approximately 40% of the total FA content in berry pulp (non-seed tissue). However, the molecular mechanisms contributing to the high accumulation of C16:1n7 in developing sea buckthorn berry pulp (SBP) remain poorly understood.

**Results:**

We identified 1737 unigenes associated with lipid metabolism through RNA-sequencing analysis of the four developmental stages of berry pulp in two sea buckthorn lines, ‘Za56’ and ‘TF2–36’; 139 differentially expressed genes were detected between the different berry pulp developmental stages in the two lines. Analyses of the FA composition showed that the C16:1n7 contents were significantly higher in line ‘Za56’ than in line ‘TF2–36’ in the mid-late developmental stages of SBP. Additionally, qRT-PCR analyses of 15 genes involved in FA and triacylglycerol (TAG) biosynthesis in both lines revealed that delta9-ACP-desaturase (*ACP-Δ9D*) competed with 3-ketoacyl-ACP-synthase II (*KASII*) for the substrate C16:0-ACP and that *ACP-Δ9D* and delta9-CoA-desaturase (*CoA-Δ9D*) gene expression positively correlated with C16:1n7 content; *KASII* and fatty acid elongation 1 (*FAE1*) gene expression positively correlated with C18:0 content in developing SBP. Specifically, the abundance of *ACP-Δ9D* and *CoA-Δ9D* transcripts in line ‘Za56’, which had a higher C16:1n7 content than line ‘TF2–36’, suggests that these two genes play an important role in C16:1n7 biosynthesis. Furthermore, the high expressions of the glycerol-3-phosphate dehydrogenase (*GPD1*) gene and the WRINKLED1 (WRI1) transcription factor contributed to increased biosynthesis of TAG precursor and FAs, respectively, in the early developmental stages of SBP, and the high expression of the diacylglycerol O-acyltransferase 1 (*DGAT1*) gene increased TAG assembly in the later developmental stages of SBP. Overall, we concluded that increased *ACP-Δ9D* and *CoA-Δ9D* levels coupled with decreased *KASII* and *FAE1* activity is a critical event for high C16:1n7 accumulation and that the coordinated high expression of *WRI1*, *GPD1*, and *DGAT1* genes resulted in high oil accumulation in SBP.

**Conclusion:**

Our results provide a scientific basis for understanding the mechanism of high C16:1n7 accumulation in berry pulp (non-seed tissue) and are valuable to the genetic breeding programme for achieving a high quality and yield of SBP oil.

**Electronic supplementary material:**

The online version of this article (10.1186/s12870-019-1815-x) contains supplementary material, which is available to authorized users.

## Background

Sea buckthorn is a perennial shrub or small tree belonging to the genus *Hippophae* L. in the family Elaeagnaceae [1]. Sea buckthorn is one of the nutritionally and ecologically most important woody oil crops and has gained popularity worldwide due to berry pulp oil, which contains uniquely bioactive compounds [2]. SBP is a good source of edible oil. Bioactive oils (2–38%) with high levels of the uncommon fatty acid (FA) palmitoleic acid (approximately 40%) [2–4], carotenoids, flavonol glycosides, and tocopherol [5–7], can be extracted from the orange or red berry pulp.

Plant oils, mainly composed of triacylglycerols (TAGs), are an essential component of human diets. The common vegetable oils extracted from palm, soybean, rapeseed and sunflower contain five main FAs: palmitic (C16:0), stearic (C18:0), oleic (C18:1), linoleic (C18:2) and α-linolenic (C18:3) acids. In addition, many uncommon FAs are synthesized. Palmitoleic acid (*cis*-Δ9–16:1 or C16:1n7) is an important unusual omega-7 FA that has good oxidative stability for food production and provides human health benefits, such as increasing cell membrane fluidity and tissue regeneration, reducing inflammation, protecting the cardiovascular system, inhibiting oncogenesis, and treating hypertriglyceridaemia, with the additional benefits of decreasing low-density lipoprotein cholesterol and increasing high-density lipoprotein cholesterol levels [8–11]. C16:1n7 is rarely synthesized in common oil crops, and few plants are known to accumulate C16:1n7. Those that do include cat’s claw (*Doxantha unguis-cati*), which contains 64% C16:1n7 [12], followed by Tasmanian waratah (*Telopea truncata*) (45%) and macadamia nuts (*Macadamia integrifolia*) (30%) [13, 14]. However, the seeds of these plants are the source of C16:1n7, and no reports have addressed C16:1n7 biosynthesis and accumulation mechanisms in SBP and non-seed tissues.

FA biosynthesis and TAG accumulation in plant seeds involve multiple subcellular organelles and several enzymatic reactions [15]. The first step in FA biosynthesis is the conversion of acetyl coenzyme A (acetyl-CoA) into malonyl-acyl carrier protein (ACP) by acetyl-CoA carboxylase (ACC, EC 6.4.1.2 from KEGG database) and ACP-S-malonyl transferase (MAT). Malonyl-ACP is elongated to palmitoyl (C16:0)-ACP by the addition of two carbons at a time (six cycles of four reactions) via the fatty acid synthase (FAS) complex [16], and C16:0-ACP is the converted to C18:0-ACP and C16:1n7-ACP by 3-ketoacyl-ACP-synthase II (KAS II, EC 2.3.1.179) and delta-9 desaturase (Δ9D, EC 1.14.19.1), respectively. In plastids, these free FAs (C16:0, C16:1n7, C18:0, and C18:1n9) released from ACP by thioesterases are subsequently exported to the endoplasmic reticulum (ER) in the form of acyl-coenzyme A (acyl-CoA) esters [17, 18]. Acyl-CoAs can be further desaturated to C18:1n7, C18:2n6, and C18:3n3 in the ER by fatty acid elongation 1 (FAE1, EC 2.3.1.199) [8], fatty acid desaturase 2 (FAD2, EC 1.14.19.6), and fatty acid desaturase 3 (FAD3, EC 1.14.19.25), respectively, or utilized in the acylation of glyceraldehyde 3-phosphate (G3P), which is a primary substrate for TAG biosynthesis, and can be produced via a reaction catalysed by glycerol-3-phosphate dehydrogenase (GPD1, EC 1.1.1.8) during glycerol synthesis [19]. There are two TAG biosynthesis pathways. The Kennedy pathway is a major pathway for TAG biosynthesis in plants. In this process, the assembly of TAG from G3P and acyl-CoA involves three sequential acylations; the first two acylations of G3P are catalysed by glycerol-3-phosphate acyltransferase (GPAT, EC 2.3.1.15) and lysophosphatidic acid acyltransferase (LPAT, EC 2.3.1.51), respectively, followed by dephosphorylation by phosphatidate phosphatase (LPIN, EC 3.1.3.4) to produce DAG, and the third and final acylation is catalysed by diacylglycerol O-acyltransferase (DGAT, EC 2.3.1.20) to produce TAG [18]. In the second pathway, phosphatidylcholine (PC) is formed first, and its acyl residues are further desaturated. The choline phosphate residue is then liberated by hydrolysis, and the corresponding DAG is acylated. The phospholipid: diacylglycerol acyltransferase (PDAT, EC 2.3.1.158) results in the conversion of DAG to TAG [20]. Final TAG storage occurs in ER-derived oil bodies [19]. In addition, transcription factors (TFs) including WRINKLED1 (WRI1), LEAFY COTYLEDON (LEC), and FUSCA 3 (FUS3) positively regulate the expression of genes involved in FA biosynthesis and control plant oil levels [21–23]. The mechanism of normal FA biosynthesis and accumulation has been widely understood in model plant seeds in previous studies, such as *Arabidopsis*, *Glycine max* and *Brassica napus* [24, 25], but the regulatory mechanism of unusual omega-7 FAs (such as C16:1n7) biosynthesis and TAG accumulation in sea buckthorn, especially in non-seed tissue, is poorly understood.

To identify genes related to C16:1n7 biosynthesis and TAG accumulation in SBP, we first generated a comprehensive transcriptome of berry pulp in four developmental stages from two lines, ‘Za56’ and ‘TF2–36’, that displayed substantial changes in oil content and FA composition. We identified 16 sets of differentially expressed genes (DEGs) during the development of berry pulp and identified the transcriptional patterns of DEGs involved in lipid metabolism. Furthermore, expression analysis of key genes in FA biosynthesis and TAG accumulation revealed that the high expression of *ACP-Δ9D* and *CoA-Δ9D* coupled with the downregulation of *KASII* and *FAE1* is a critical event for high C16:1n7 accumulation, and the coordinated high expression of *GPD1* and *WRI1* in the mid-early developmental stages and *DGAT1* in the mid-late stages increased TAG assembly in SBP. These results provide a scientific basis for understanding the mechanism of C16:1n7 biosynthesis and accumulation in berry pulp (non-seed tissue) and are valuable to the genetic breeding programme for achieving a high quality and yield of SBP oil.

### Oil extraction and analysis

Total oils were extracted from SBP as described previously [26]. Briefly, 0.3 g of dried pulp powder (*m*_1_) was homogenized in 6 mL chloroform-methanol (2:1, v/v) for 2 min. The mixtures were sonicated in an ultrasonic bath for 30 min, centrifuged and filtered. The solid residues were re-suspended in 4 mL chloroform-methanol (2:1, v/v), homogenized for 3 min and filtered. A volume of 1 mL 0.88% KCl was added to the combined filtrates, and the mixtures were mixed thoroughly by vortexing and were then centrifuged. The lower phase containing the purified oils (*m*_2_) was collected and evaporated under nitrogen. The oil content was calculated as follows: oil content (%) = *m*_2_/*m*_1_ × 100%.

### Fatty acid methyl ester (FAME) and gas chromatography time-of-flight mass spectrometry (GC-TOF/MS) analyses of berry pulp

The FA composition was determined by FAMEs extraction with boron trifluoride in methanol as a catalyst according to previous studies [27, 28]. Briefly, 0.2 g of the dried powder samples was transferred into a glass test tube, followed by the addition of 2 mL n-hexane and 5 mL methanol-potassium hydroxide solution (1 M). The mixture was placed in a vibrating water bath at 60 °C for 30 min. After the reaction was complete, 10 mL boron trifluoride in methanol was added to the mixture, and the samples were left at 60 °C for 30 min. Two millilitres of saturated sodium chloride solution and 2 mL n-hexane were added, and FAMEs were then extracted by vigorous shaking for approximately 1 min. Following centrifugation, the aliquots were dried with anhydrous sodium sulfate, and the top layer was transferred into a vial, which was flushed with nitrogen; the vials were stored at − 20 °C until analysed by gas chromatography. The analyses were conducted in triplicate.

GC-TOF/MS analysis of FAMEs was performed on a Clarus 680 GC coupled to an AxION iQT TOF/MS system (PerkinElmer, Shelton, USA). The system was equipped with an Agilent J&W DB-23 capillary column (60 m × 0.25 mm × 0.25 μm). Helium was used as a carrier gas (flow rate: 1 mL·min^− 1^). The injector was operated in split mode (1:20). The inlet and transfer line temperatures were 230 °C and 215 °C, respectively. The temperature program was as follows: the initial temperature of 50 °C was raised by 15 °C·min^− 1^ to 200 °C, increased by 3 °C·min^− 1^ to 215 °C and finally increased to 230 °C for 10 min. For the detection of the mass spectrum, the temperature of the ion source was 230 °C. The operation parameters were an electron ionization (EI) ion source, electron energy of 70 eV, electron multiplier of 1.5 kV, solvent delay of 5 min, scanning range of 45–400 amu, and injection volume of 1 μL using the full-scan mode. FAs were identified by comparison with the retention times in the total ion chromatography (TIC) spectra and the mass-to-charge ratio (m/z) in the mass spectra of 37 FAME standards (Sigma-Aldrich, Steinheim, Germany). The FA composition was expressed as the weight percentage of each FA in the total FA content.

### Total RNA extraction, cDNA library construction and RNA-seq analysis

Total RNA was isolated from berry pulp in four developmental stages using a TRIzol RNA Extraction Kit (Invitrogen, Carlsbad, CA, USA). Total RNA was quantified and qualified by 1% gel electrophoresis, a NanoDrop (Thermo Fisher Scientific Inc.) and an Agilent 2100 bioanalyser (Agilent Technologies, Palo Alto, CA, USA). RNA samples with an A260/A280 ratio between 1.9 and 2.1, an A260/A230 ratio between 2.0 and 2.5 and a RIN (RNA integrity number) of ≥8.0 were processed for further analysis.

Total RNA from three biological replicates of each sample was used for cDNA preparation. Next-generation sequencing library preparations were constructed according to the manufacturer’s protocol (NEBNext Ultra™ RNA Library Prep Kit for Illumina). mRNA enrichment, fragment interruption, adapter addition, size selection and PCR amplification were performed by GENEWIZ Inc. (Beijing, China), and RNA-seq was conducted by an Illumina HiSeq2500 system. Briefly, mRNA was purified from 3 μg total RNA from berry pulp at each developmental stage from the two lines using oligo (dT) magnetic beads. Next, using these short fragments as templates, first-strand cDNA synthesis was carried out with random primers. Third, the ends of double-stranded cDNA fragments were further modified with DNA polymerase. After the end-repair process and adapter ligation, the products were enriched by PCR to construct the final cDNA library. The cDNA library was examined using an Agilent 2100 bioanalyser [29]. Finally, the eight berry pulp samples, from the four developmental stages of the two lines, were sequenced.

### Assembly and functional annotation of unigenes

Eight RNA-seq libraries were constructed, and de novo transcriptome libraries were assembled from all samples. Ambiguous nucleotides (N) and low-quality bases in raw reads were removed based on the Q20, Q30, N and GC parentages to obtain clean reads. De novo assembly was performed using Trinity software r2013-02-25 [30]. The total and average lengths of the assembled contigs were important criteria for assessing transcriptome quality. Unigenes were defined after removing redundant and short contigs from the assembly.

Reads were mapped against the assembled de novo transcriptome using Bowtie2 [31]. All unigenes from sea buckthorn were aligned using BLAST to identify homologous genes and to the non-redundant protein (nr), Clusters of Orthologous Groups (COG), Swiss-Prot (an annotated protein sequence database) and Kyoto Encyclopedia of Genes and Genomes (KEGG) databases [32] using Blastx algorithms with a threshold E-value of ≤10^− 5^. The unigenes were mapped to the KEGG metabolic pathway database to elucidate the complex biological behaviours of unigenes using KEGG Automatic Annotation Server (KASS) [33]. Using the COG database, orthologous gene products were classified, and the functions of unigenes were predicted.

### Identification of DEGs involved in FA biosynthesis and TAG accumulation

DEGs were identified from the unigenes for digital gene expression profile analysis, which was performed with a modified procedure using a rigorous algorithm. The normalization and calculation of unigene expression was performed by the fragments per kilobase of exon model per million mapped fragments (FPKM) method. A *p* value of < 0.05 and an absolute value of the log_2_ (fold change) of > 1 were the thresholds for significant differences in gene expression.

The significant DEGs involved in FA biosynthesis and TAG accumulation were screened based on alignments against the lipid metabolism pathways in the KEGG database. We first compared transcriptome differences among the four developmental stages of pulp in sea buckthorn berries specifically by analysing the transcript profiles of multigenes (*ACC*, *KAR*, *FATA*, *KASII*, *ACP-Δ9D*, *CoA-Δ9D*, *FAE1*, *FAD2*, *FAD3*, *GPD1, GPAT*, *LPAT*, *DGAT1*, *PDAT*, and *WRI1*) associated with C16:1n7 biosynthesis and TAG accumulation in berry pulp.

### qRT-PCR analysis

The RNA samples used for qRT-PCR analysis were the same as those used for the RNA-seq, oil content and FA composition analyses. The first-strand cDNA used for qRT-PCR was synthesized from RNAs using a PrimeScript RT reagent kit with gDNA Eraser (TaKaRa, Dalian, China).

Primer pairs were designed according to the selected multigenes coding for FA and TAG biosynthesis enzymes/proteins using Primer 5.0 software (Additional file 1: Table S1). The *UBQ5* gene was used as a reference gene for quantitative gene expression [4]. The qRT-PCR reactions were performed in 96-well plates using an ABI 7500 Real-Time PCR system (Applied Biosystems, Foster, United States) and a SYBR Premix Ex Taq II Kit (TaKaRa, Dalian, China) according to the manufacturer’s instructions. qRT-PCR was carried out on three replicates per sample, and the relative expression level of each gene was calculated by the 2^*-ΔΔCt*^ method [34].

## Results

### Characterization of oil content and FA composition during berry pulp development in sea buckthorn

**Fig. 1:**
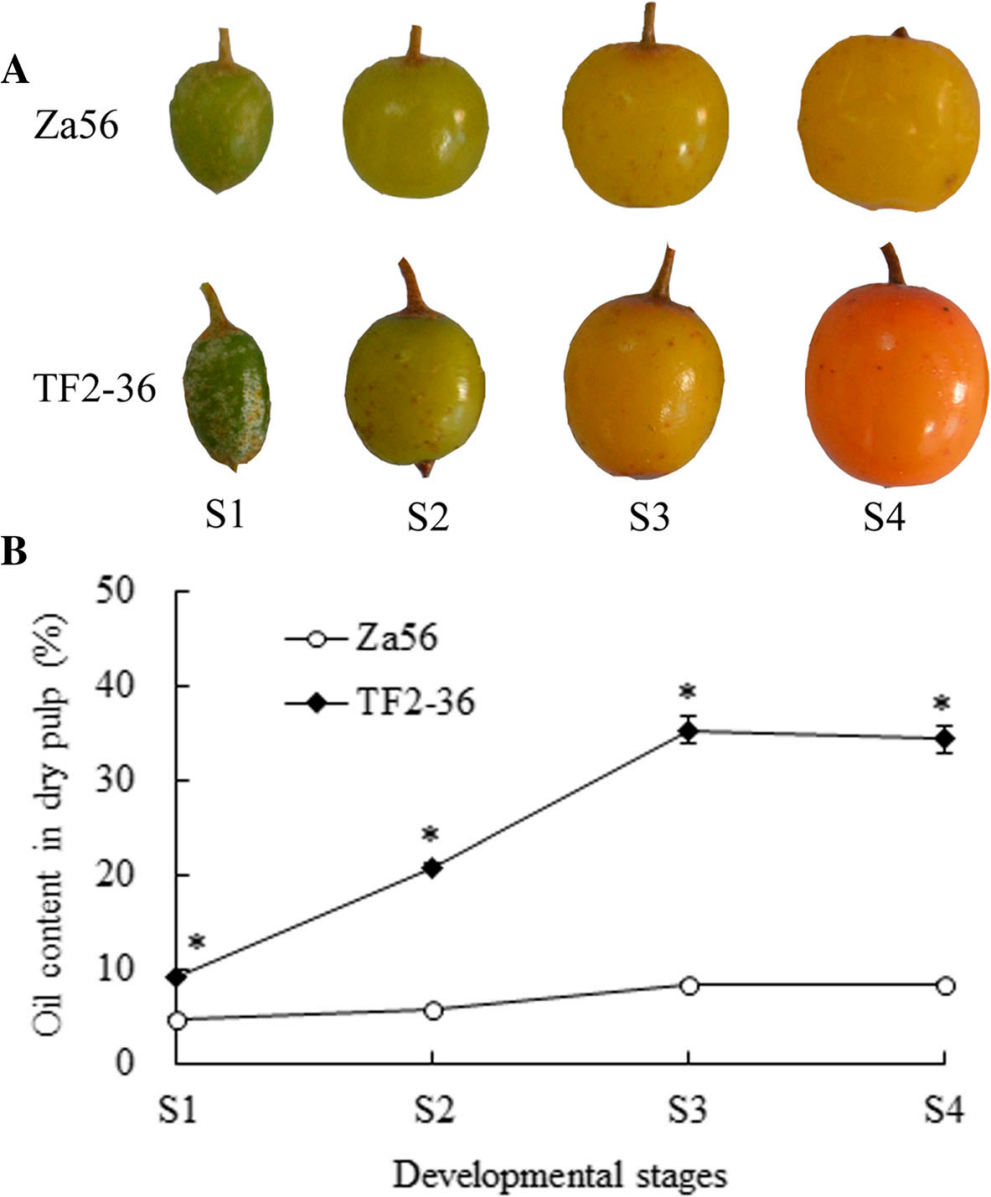
Phenotypic observations and oil content in the developing berry pulp of the two sea buckthorn lines ‘Za56’ and ‘TF2–36’. **a** The developmental progress of fruits from lines ‘Za56’ and ‘TF2–36’ (S1–S4). S1, S2, S3 and S4 indicate the developmental stage of berry pulp collected on July 6, July 28, August 19 and September 10, respectively. **b** Oil content was measured at four developmental stages during pulp development in lines ‘Za56’ and ‘TF2–36’. * indicate significant differences of data between the two lines at the same developmental stage at the level of 0.05

Oil content and FA composition across four developmental stages (S1, S2, S3, and S4) of SBP lines, ‘TF2–36’ and ‘Za56’ (Fig. 1a), were explored. The oil contents in the developing berry pulp of line ‘TF2–36’ exhibited rapid accumulation from 9.31% at S1 stage to 35.42% at S3 stage and were significantly higher than those in line ‘Za56’ (4.84–8.48%). During S3 to S4, the SBP oil contents of both lines remained stable (Fig. 1b).

**Fig. 2:**
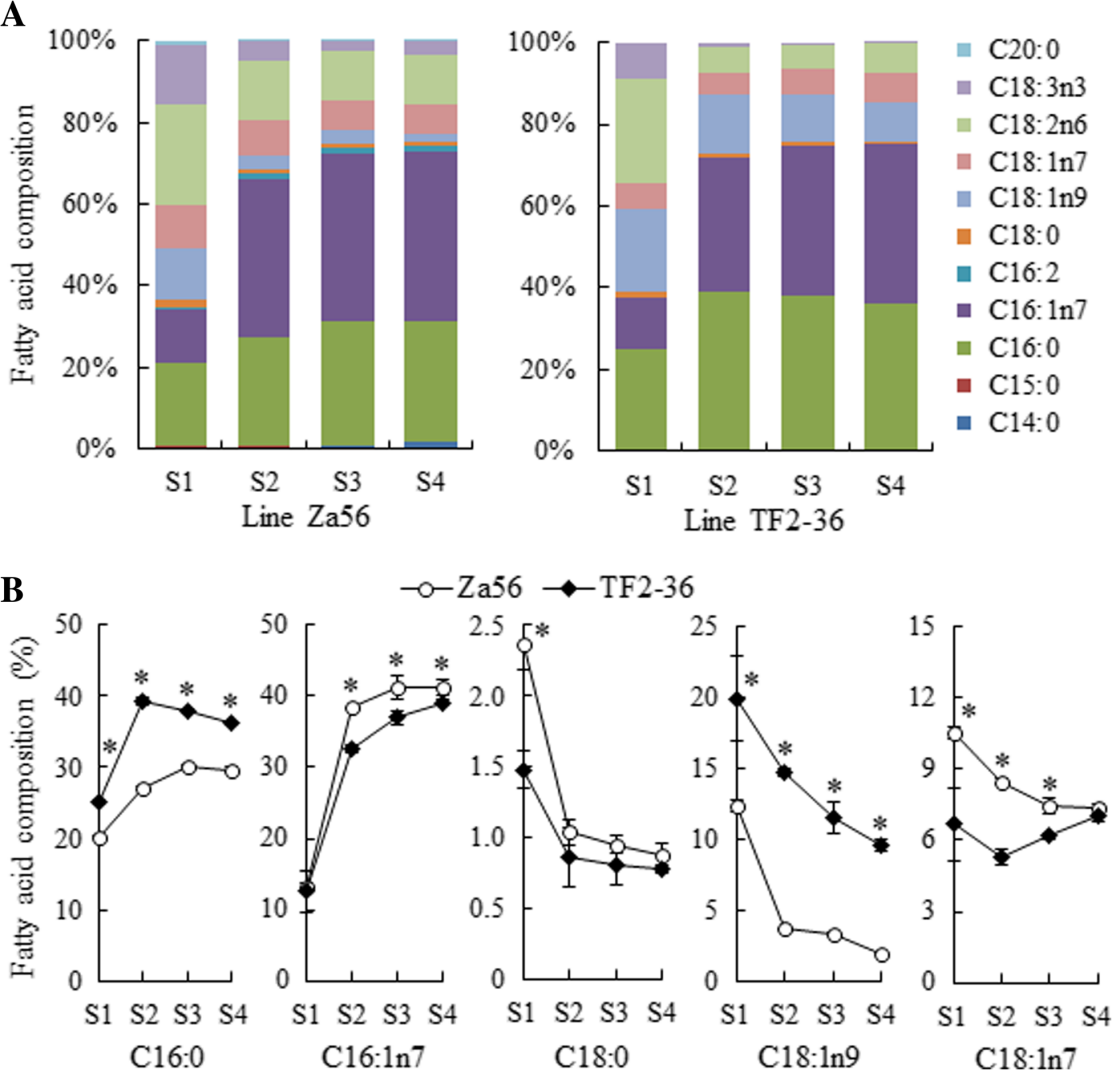
FA composition in SBP from lines ‘Za56’ and ‘TF2–36’ at four developmental stages. **a** Changes in the composition of various FAs in each line. **b** Comparison of five major FAs between two lines. The error bars indicate the standard deviations of three biological replicates. * indicate significant differences of FA composition between the two lines at the same developmental stages at the level of 0.05

To reveal the FA composition in SBP, we performed GC-TOF/MS analyses to measure the FA composition at four developmental stages. Eleven and seven kinds of FAs were measured in the berry pulp from lines ‘Za56’ and ‘TF2–36’, respectively. C16:0, C16:1n7, C18:0, oleic (C18:1n9), vaccenic (C18:1n7), C18:2n6 and C18:3n3 were identified in both lines; four other FAs (myristic, pentadecanoic, hexadecadienoic and arachidic acids) were also detected in the berry pulp from line ‘Za56’ (Fig. 2a and Additional file 2: Table S2).

Of the saturated fatty acids (SFAs), C16:0 was the most abundant in SBP, accounting for the highest (30.21% in line ‘Za56’ and 39.22% in line ‘TF2–36’) percentage of all FAs; furthermore, the C16:0 content was higher in line ‘TF2–36’ than in line ‘Za56’ at every stage (Fig. 2b). However, the C18:0 content in line ‘TF2–36’ were significantly lower than the C16:0 content in line ‘TF2–36’ and lower than the C18:0 content in line ‘Za56’ in developing berry pulp. The monounsaturated fatty acid (MUFA) (C16:1n7, C18:1n9, and C18:1n7) contents, accounting for 36.19–51.91% and 39.17–55.68% of all FAs in lines ‘Za56’ and ‘TF2–36’, respectively, were much higher than those of SFAs and polyunsaturated fatty acids (PUFAs) in each line for all four berry pulp developmental stages (Fig. 2a). In particular, the C16:1n7 content in both lines was predominant among the MUFAs, accounting for 13.31–41.30% in line ‘Za56’ and 12.57–39.03% in line ‘TF2–36’ and increasing during berry pulp development, and the C16:1n7 contents in line ‘Za56’ were higher than those in line ‘TF2–36’ from S2 to S4 (Fig. 2b and Additional file 2: Table S2). The contents of C18:1n9 in line ‘Za56’ (1.98–12.35%) were significantly lower than those in line ‘TF2–36’ (9.60–19.92%) and decreased from S1 to S4, but the C18:1n7 contents in line ‘Za56’ (7.34–10.53%) were higher than those in line ‘TF2–36’ (5.32–7.05%) (Fig. 2b). For the PUFAs, the contents of C18:2n6 and C18:3n3 decreased greatly from S1 to S3, and these two FAs were much more abundant in developing berry pulp from line ‘Za56’ than from line ‘TF2–36’ (Fig. 2a and Additional file 2: Table S2).

### Sequencing, de novo transcriptome assembly and functional annotation

**Fig. 3:**
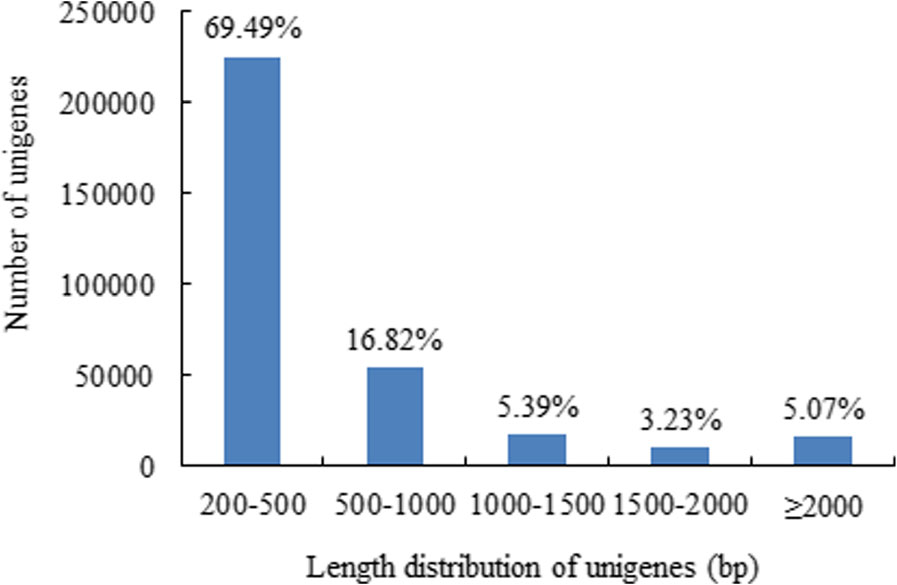
Length distributions of assembled unigenes

To obtain an overview of the sea buckthorn transcriptome in developing berry pulp and identify key candidate genes involved in lipid biosynthesis and accumulation in berry pulp (non-seed tissue), we performed transcriptome sequencing of berry pulps in four developmental stages (S1, S2, S3 and S4) from two sea buckthorn lines, ‘Za56’ and ‘TF2–36’. After removing adapter sequences, duplicated sequences, ambiguous reads and low-quality reads, a total of 397,823,136 clean reads were generated; 99.27–99.58% of bases had a Q of ≥20, and the reads had a 41.1–41.8% GC content (Table 1). The reads in each library were assembled into unigenes using the program Trinity. As a result, 323,881 unigenes were generated (≥ 200 bp), with an average size of 593.25 bp and an N50 length of 856 bp. In total, 98,829 unigenes (30.51%) were longer than 500 bp (Fig. 3). The mapping rates of clean reads mapped to unigenes were 54.84–66.25% (Table 1).

**Table 1:** Quality analysis of clean reads of RNA-seq

Sample	Stage	Length of read	Number of reads	Total bases	Q20 (%)	Q30 (%)	GC (%)	N (ppm)	Mapped (%)
Line Za56 pulp	S1	142.36	42,719,700	6,081,446,055	99.33	97.53	41.10	2.24	60.86
	S2	139.48	41,463,302	5,783,400,256	99.27	97.21	41.56	3.23	54.84
	S3	142.98	43,184,998	6,174,487,450	99.29	97.40	41.48	2.04	64.66
	S4	143.46	55,867,418	8,014,903,918	99.53	98.05	41.36	8.45	64.71
Line TF2–36 pulp	S1	144.18	48,471,784	6,988,472,233	99.58	98.25	41.80	5.70	66.25
	S2	143.69	60,647,368	8,714,191,825	99.57	98.21	41.21	5.81	64.87
	S3	143.59	59,674,434	8,568,834,801	99.54	98.08	41.41	4.20	65.35
	S4	143.01	45,794,132	6,548,950,087	99.56	98.14	41.35	4.28	63.36

There were 79,413 unigenes (24.52%) annotated in the nr database, 69,924 unigenes (21.59%) annotated in the COG database, 24,456 unigenes (7.55%) annotated in the Gene Ontology (GO) database, 99,916 unigenes (30.85%) annotated in the Swiss-Prot database and 28,579 unigenes (8.82%) annotated in the KEGG database. Thus, a total of 122,305 (37.76%) unigenes were annotated in one or more public databases (Table 2 and Additional file 3: Table S3), and 15,997 unigenes were annotated in all four databases.

**Table 2:** Functional annotation of the sea buckthorn transcriptome

Public database	Number of unigenes	Percentage (%)
nr	79,413	24.52
COG	69,924	21.59
GO	24,456	7.55
Swiss-Prot	99,916	30.85
KEGG	28,579	8.82
Total	122,305	37.76

**Fig. 4:**
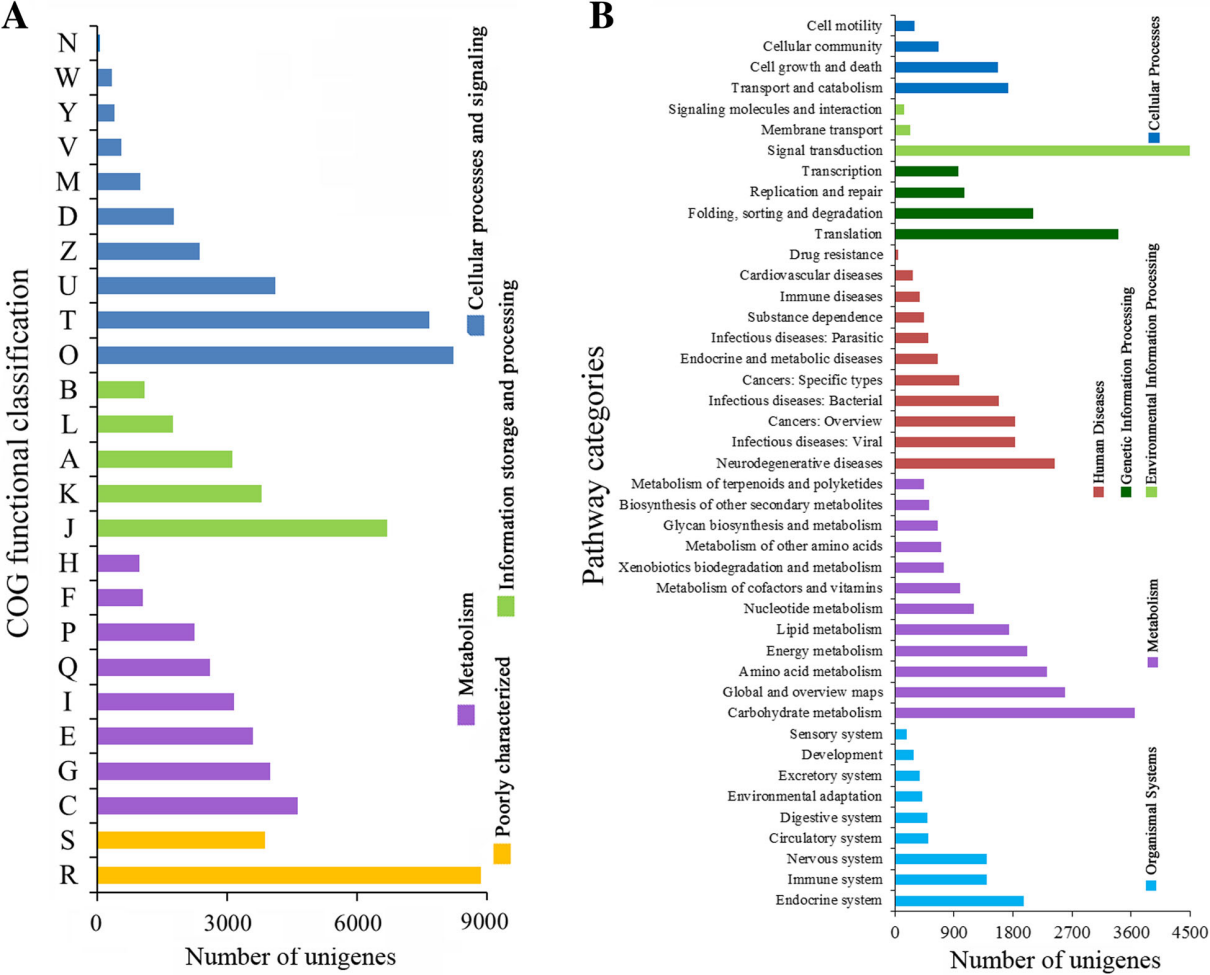
Functional classifications and annotation of unigenes. **a** COG classification: A, RNA processing and modifications; B, Chromatin structure and dynamics; C, Energy production and conversion; D, Cell cycle control, cell division, chromosome partitioning; E, Amino acid transport and metabolism; F, Nucleotide transport and metabolism; G, Carbohydrate transport and metabolism; H, Coenzyme transport and metabolism; I, Lipid transport and metabolism; J, Translation, ribosomal structure and biogenesis; K, Transcription; L, Replication, recombination and repair; M, Cell wall/membrane/envelope biogenesis; N, Cell motility; O, Posttranslational modification, protein turnover, chaperones; P, Inorganic ion transport and metabolism; Q, Secondary metabolites biosynthesis, transport and catabolism; R, General function prediction only; S, Function unknown; T, Signal transduction mechanisms; U, Intracellular trafficking, secretion, and vesicular transport; V, Defence mechanisms; W, Extracellular structures; Y, Nuclear structure; Z, Cytoskeleton. **b** KEGG classifications

Some unigenes were annotated with multiple COG functions; therefore, 77,713 functional annotations were assigned to 25 COG clusters, which were grouped into four larger categories, namely, “cellular processes and signalling” (26,396 unigenes, 33.97%); “information storage and processing” (16,402, 21.11%); “metabolism” (22,202, 28.57%), containing 3153 unigenes involved in “lipid transport and metabolism” (Fig. 4a); and “poorly characterized” (12,713, 16.36%).

The KEGG pathway database with the KAAS was used to predict the metabolic network of sea buckthorn. Using Blast2GO, all unigenes were mapped against the KEGG database, resulting in 28,579 unigenes grouped into 374 KEGG pathways and 43 pathway categories, which were grouped into six groups (Fig. 4b). The groups most enriched in unigenes were metabolism (17,572 unigenes). We focused on the “lipid metabolism” category, containing 1737 unigenes grouped into 17 pathways. The maximum number of unigenes involved in glycerophospholipid metabolism (ko00564) was 342; 222 unigenes were involved in glycerolipid metabolism (ko00561), and 141 unigenes were involved in FA biosynthesis (ko00061). Furthermore, 131 were related to the biosynthesis of unsaturated fatty acids (ko01040), 83 were in the FA elongation pathway (ko00062) and 189 were in the FA degradation pathway (ko00071).

### Identification of DEGs involved in palmitoleic acid biosynthesis and TAG accumulation in SBP

**Fig. 5:**
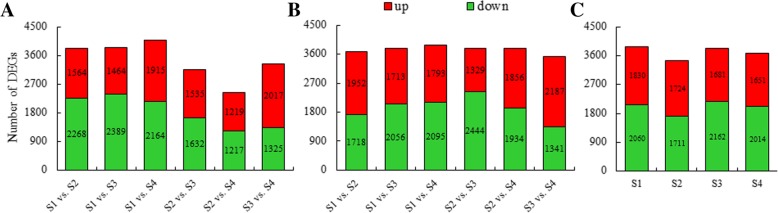
Numbers of upregulated and downregulated DEGs in the two sea buckthorn lines. **a** Numbers of DEGs in line ‘Za56’ by pairwise comparisons of four developmental stages. **b** Numbers of DEGs in line ‘TF2–36’ by pairwise comparisons of four developmental stages. **c** Numbers of DEGs between the two lines at the same developmental stage

To investigate the regulatory mechanism of FAs and TAG biosynthesis, especially the high accumulation of C16:1n7 in berry pulp (non-seed tissue), lipid metabolism pathways with unigenes identified in the SBP transcriptome were evaluated. We compared the normalized read counts of the unigenes and identified significant DEGs (*p* value < 0.05) among the developing SBP of lines ‘Za56’ and ‘TF2–36’. The significant DEGs were compared between the libraries of two different developmental stages (16 pairwise comparison groups, in Additional file 4: Table S4). A total of 30,530 downregulated and 27,430 upregulated DEGs were identified among the 16 pairwise comparison groups (Fig. 5). Compared to the number of DEGs in S1, the number of DEGs increased from S2 to S4. C16:1n7 and oils mostly accumulated in SBP during the period from S2 to S4 (Figs. 1b and 2b). In the S1 vs. S4 comparison for each line (Figs. 5a, b), we found the highest number of DEGs (3888 and 4079 in lines ‘Za56’ and ‘TF2–36’, respectively). A total of 3890, 3435, 3843 and 3665 DEGs were identified in the four pairwise comparison groups (S1, S2, S3 and S4, respectively) between lines ‘Za56’ and ‘TF2–36’ (Fig. 5c).

**Fig. 6:**
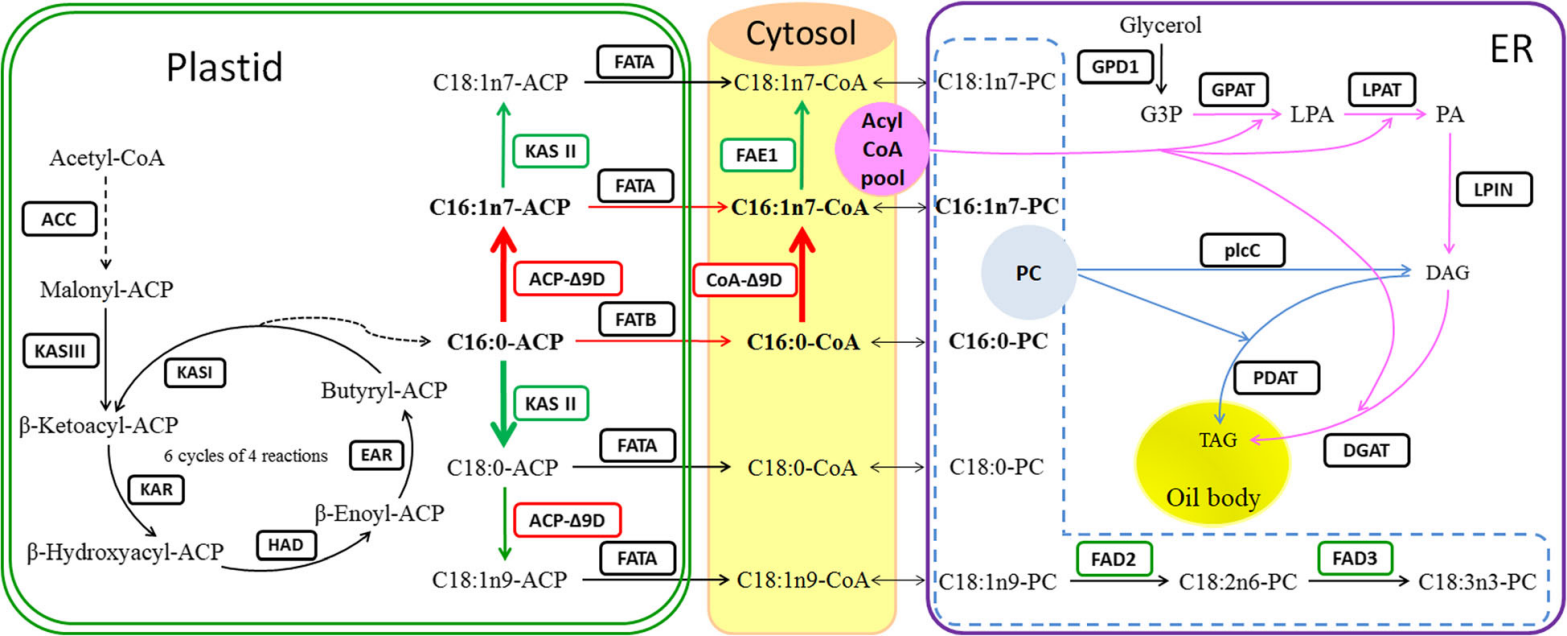
Schematic diagram representing palmitoleic acid biosynthesis and TAG accumulation in SBP. The bold arrows in red indicate the metabolic flux from C16:0-ACP to C16:1n7-TAG. The red (upregulation) and green (downregulation) boxes indicate the key genes in C16:1 biosynthesis and its accumulation in TAG. The violet arrows indicate the Kennedy pathways in TAG assembly. The blue arrows indicate the phosphatidylcholine acyl pathways in TAG assembly. The enzymes are shown in boxes and abbreviated as follow: ACC, acetyl-CoA carboxylase; KAS, 3-ketoacyl-ACP synthase (KAS I, KAS II, KAS III); KAR, 3-ketoacyl-ACP reductase; HAD, β-hydroxyacyl-ACP dehydrase; EAR, enoyl-ACP reductase; FATA, acyl-ACP thioesterase A; FATB, acyl-ACP thioesterase B; Δ9D, delta-9 desaturase; FAE1, fatty acid elongation 1; FAD2, fatty acid desaturase 2; FAD3, fatty acid desaturase 3; GPD1, glycerol-3-phosphate dehydrogenase; GPAT, glycerol-3-phosphate O-acyltransferase; LPAT, lysophosphatidic acid acyltransferase; LPIN, phosphatidate phosphatase; DGAT, diacylglycerol O-acyltransferase; plcC, phospholipase C; PDAT, phopholipid:diacyglycerol acyltransferase. The names of key intermediates are abbreviated as follows: G3P, glycerol-3-phosphate; LPA, lysophosphatidic acid; PA, phosphatidic acid; DAG, diacylglycerol; TAG, triacylglycerol

**Fig. 7:**
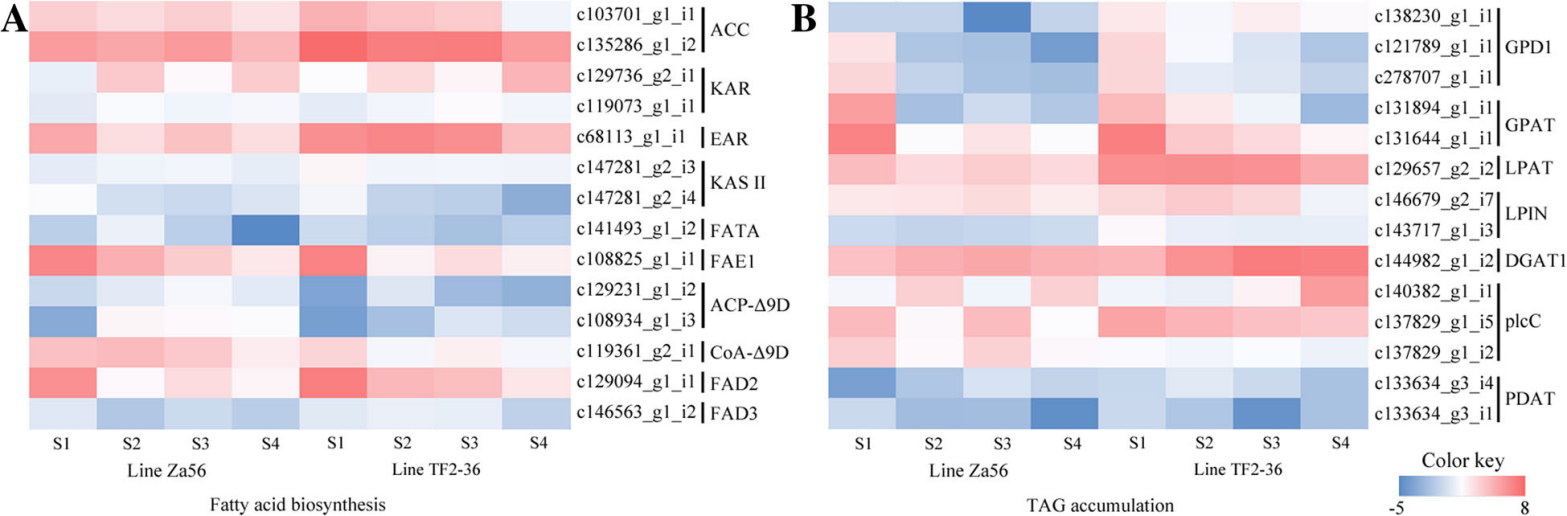
Heat maps of unigenes involved in FA biosynthesis (**a**) and TAG accumulation (**b**) in SBP. The expression value (in FPKM) for the unigenes during berry pulp development in both lines was log_2_ transformed, and the total FPKM value was greater than 20

Based on alignments against the KEGG pathway analysis, 139 DEGs (42 functional genes) were related to lipid metabolism (Table 3**)**. Of these, 65 were involved in FA biosynthesis pathways in plastids, and 74 were associated with TAG biosynthesis (Additional file 5: Table S5). ACC is a key enzyme of FA precursor biosynthesis. The FPKMs of *ACC* (two homologous DEGs) were first upregulated from S2 to S3 and then downregulated from S3 to S4 in developing SBP of line ‘Za56’, and the total FPKM of line ‘Za56’ was much lower than that in line ‘TF2–36’ (Fig. 7a), which could provide more FA substrates for the assembly of TAG. To investigate the high level of C16:1n7 accumulation in SBP, we focused on several key enzymes related to C16:1n7 biosynthesis, which involves desaturation by two distinct classes of *Δ9D* genes, designated *ACP-Δ9D* and *CoA-Δ9D* (Fig. 6). The *ACP-Δ9D* gene catalyses the conversion of C16:0-ACP to C16:1n7-ACP, and the *CoA-Δ9D* gene catalyses the conversion of C16:0-CoA to C16:1n7-CoA, which have two and one homologous DEGs, respectively, and a total FPKM value of greater than 20 (Fig. 7a). The FPKMs of these three unigenes increased and then decreased, and the expression profiles of these genes correlated with the C16:1n7 content in both lines. In particular, the peak expression of these genes in line ‘Za56’, with a high C16:1n7 content, was higher than that in line ‘TF2–36’, with a low C16:1n7 content, during berry pulp development (Figs. 2b and 7a). Notably, two homologous DEGs of *KASII*, catalysing the conversion of C16:0-ACP to C18:0-ACP and/or C16:1n7-ACP to C18:1n7-ACP (Fig. 6), had downregulated expression patterns with low levels in the mid-late stages (Fig. 7a), and the C18:0 contents in both lines were less than 1% (Fig. 2b). Furthermore, the downregulation of *FAE1*, elongating of C16:1n7-CoA to C18:1n7-CoA, coordinated with low expression of *KASII* (Fig. 7a), which was responsible for the low contents of C18:1n7 (Fig. 2b). The transcription of *FAD2* and *FAD3* was also downregulated (Fig. 7a). These *Δ9D* and *KASII* genes regulated the reaction direction of C16:0-ACP conversion to C16:1n7-ACP and/or C18:0-ACP, suggesting a critically synergistic regulatory mechanism involved in high C16:1n7 accumulation in SBP.

**Table 3:** Differentially expressed genes involved in fatty acid and triacylglycerol biosynthesis

Function	Enzyme		Unigene name
	Description	Abbr.	
Fatty acid biosynthesis	acetyl-CoA carboxylase	*ACC*	c103701_g1_i1, c135286_g1_i2
	long-chain acyl-CoA synthetase	*ACSL*	c128996_g1_i1, c147409_g1_i3, c128996_g1_i1, c144079_g1_i3, c141518_g1_i4, c144079_g1_i3, c251011_g1_i1, c136897_g1_i3
	delta9-ACP desaturase	*ACP-Δ9D*	c108934_g1_i3, c129231_g1_i1, c129231_g1_i2, c139467_g4_i2, c139467_g4_i3, c139467_g4_i4
	delta9-CoA desaturase	*CoA-Δ9D*	c119361_g2_i1, c141805_g5_i1, c99943_g1_i2
	3-ketoacyl-ACP synthase II	*KAS II*	c147281_g2_i4, c147281_g2_i3
	3-ketoacyl-ACP reductase	*KAR*	c74284_g1_i1, c129736_g2_i1, c119073_g1_i1, c89810_g1_i1, c74284_g1_i1
	fatty acid elongation 1	*FAE1*	c120999_g2_i1
	3-ketoacyl-ACP synthase III	*KAS III*	c142490_g1_i1
	enoyl-ACP reductase	*EAR*	c68113_g1_i1
	fatty acyl-ACP thioesterase A	*FATA*	c141493_g1_i2
	fatty acid desaturase 2	*FAD2*	c129094_g1_i1, c108838_g1_i1
	fatty acid desaturase 3	*FAD3*	c146563_g1_i2
	fatty acid desaturase 7	*FAD7*	c136784_g1_i3, c136784_g1_i5, c68263_g1_i1, c136784_g2_i5
	fatty acid desaturase 8	*FAD8*	c136784_g2_i1, c136784_g2_i4, c136784_g2_i2
	3-ketoacyl-CoA synthase	*KCS*	c127448_g1_i1, c144156_g2_i1, c144156_g3_i1, c108825_g1_i1, c144156_g3_i2, c133459_g1_i2, c144156_g2_i2
	mitochondrial trans-2-enoyl-CoA reductase	*MECR*	c128870_g1_i2
	long-chain-3-hydroxyacyl-CoA dehydratase	*PAS2*	c129950_g1_i2, c138653_g1_i3, c138653_g1_i2
	long-chain enoyl-CoA reductase	*CER10*	c144040_g1_i1
	acyl-CoA oxidase	*ACOX*	c145203_g1_i3, c126961_g1_i2, c145203_g1_i1, c142258_g1_i1, c145203_g1_i2
	acetyl-CoA acyltransferase 1	*ACAA1*	c118938_g1_i1
TAG biosynthesis	alcohol dehydrogenase	*adh*	c81969_g1_i1, c144472_g1_i1, c134965_g1_i1, c281911_g1_i1, c144736_g1_i1
	aldehyde dehydrogenase	*adhE*	c124326_g1_i1, c141482_g2_i6, c141482_g2_i2, c119206_g1_i1, c133702_g1_i1, c124761_g1_i1, c133123_g1_i1, c106355_g1_i1, c144329_g2_i1, c174192_g1_i1, c225983_g1_i1
	diacylglycerol O-acyltransferase 1	*DGAT1*	c144982_g1_i2
	diacylglycerol kinase	*DGK*	c134987_g1_i3, c141410_g1_i7, c142032_g1_i4, c142032_g1_i2, c134987_g3_i1
	1,2-diacylglycerol 3-beta-galactosyltransferase	*MGD*	c124343_g1_i1
	alpha-galactosidase	*GLA*	c125189_g1_i1
	glycerol-3-phosphate acyltransferase	*GPAT*	c131894_g1_i1, c138865_g2_i1, c131644_g1_i1, c4490_g1_i1, c127068_g1_i1
	phosphatidate phosphatase	*LPIN*	c141781_g2_i2, c146679_g2_i8, c146679_g2_i7, c143717_g1_i3
	lysophospholipid acyltransferase	*LPAT*	c129657_g2_i1, c129657_g2_i2
	acylglycerol lipase	*MGL*	c119722_g1_i2
	1-acyl-sn-glycerol-3-phosphate acyltransferase	*plsC*	c143245_g2_i1
	glycerol-3-phosphate dehydrogenase	*GPD1*	c145910_g1_i1, c138230_g1_i3, c121789_g1_i1, c278707_g1_i1, c71276_g1_i1, c138230_g1_i1
	phosphatidylglycerol phospholipase C	*plcC*	c140114_g1_i1, c126915_g1_i1, c140382_g1_i1, c137829_g1_i5, c137829_g1_i2
	phospholipase D1/2	*PLD1_2*	c146964_g1_i5, c133909_g1_i1, c146964_g1_i11, c133909_g1_i2, c146964_g1_i10, c146964_g1_i3, c146964_g1_i7, c146964_g1_i6, c146964_g1_i8
	phospholipid:diacylglycerol acyltransferase	*PDAT*	c133634_g3_i4, c133634_g3_i6, c133634_g3_i7, c133634_g3_i1
	phosphatidyl glycerophosphatase	*GEP4*	c138502_g1_i3
	phosphoethanolamine N-methyltransferase	*NMT*	c133930_g1_i1
	glycerophosphocholine phosphodiesterase	*GPCPD1*	c126428_g1_i4
	lysophospholipase II	*LYPLA2*	c144211_g2_i4
	ethanolamine phosphotransferase	*EPT1*	c146531_g1_i3
	CDP-diacylglycerol-glycerol-3-phosphate 3-phosphatidyltransferase	*PGS1*	c139239_g1_i6, c139239_g1_i9
	phosphatidylserine synthase 2	*PTDSS2*	c147897_g1_i8, c147897_g1_i7, c147897_g1_i6, c147897_g1_i4, c147897_g1_i1, c147897_g1_i2

Significant DEGs were identified based on a log_2_ (fold change) > 1 and a *p* value < 0.05

TAG assembly is an important step in oil accumulation. G3P is a primary substrate, converted into TAG by three sequential acylations by GPAT, LPAT and DGAT (Fig. 6). Nine DEGs, including 3 *GPD1*, 2 *GPAT*, 1 *LPAT*, 2 *LPIN*, and 1 *DGAT1* (the total FPKM of each transcript was greater than 20), were identified in the developing berry pulp transcriptome of sea buckthorn (Fig. 7b). *GPD1*, a rate-limiting enzyme in G3P synthesis, had the highest expression level in the mid-early stages (S1–S2), and the transcript levels in developing berry pulp from line ‘TF2–36’, with a high oil content, were higher than those in line ‘Za56’, with a low oil content (Fig. 1b). *GPAT* and *LPAT* had the highest transcript levels in S1, and the transcripts of these two genes in developing berry pulp of line ‘TF2–36’ were higher than those in line ‘Za56’. *DGAT1*, a rate-limiting enzyme in TAG assembly, had the highest expression level in the mid-late stages (S3–S4) and its transcript level was higher in line ‘TF2–36’ than in line ‘Za56’ (Fig. 7b). In addition, we identified two significant difference expression *WRI1* (c141864_g1_i1 and c141864_g1_i2) TF in de novo transcriptome library of SBP, the FPKMs of *WRI1* (c141864_g1_i1) in line ‘TF2–36’ berry pulp from S1 to S4 (30.3, 53.3, 31.2, and 10.7, respectively) were always higher than those in line ‘Za56’ (10.7, 8.2, 9.8, and 5.0, respectively). These results indicated that the high expression of the multigenes (*GPD1*, *GPAT*, *LPAT*, *LPIN*, *DGAT1*, and *WRI1*) contributed to TAG biosynthesis and accumulation.

### qRT-PCR analysis reveals differential transcript abundances during berry pulp development in sea buckthorn

**Fig. 8:**
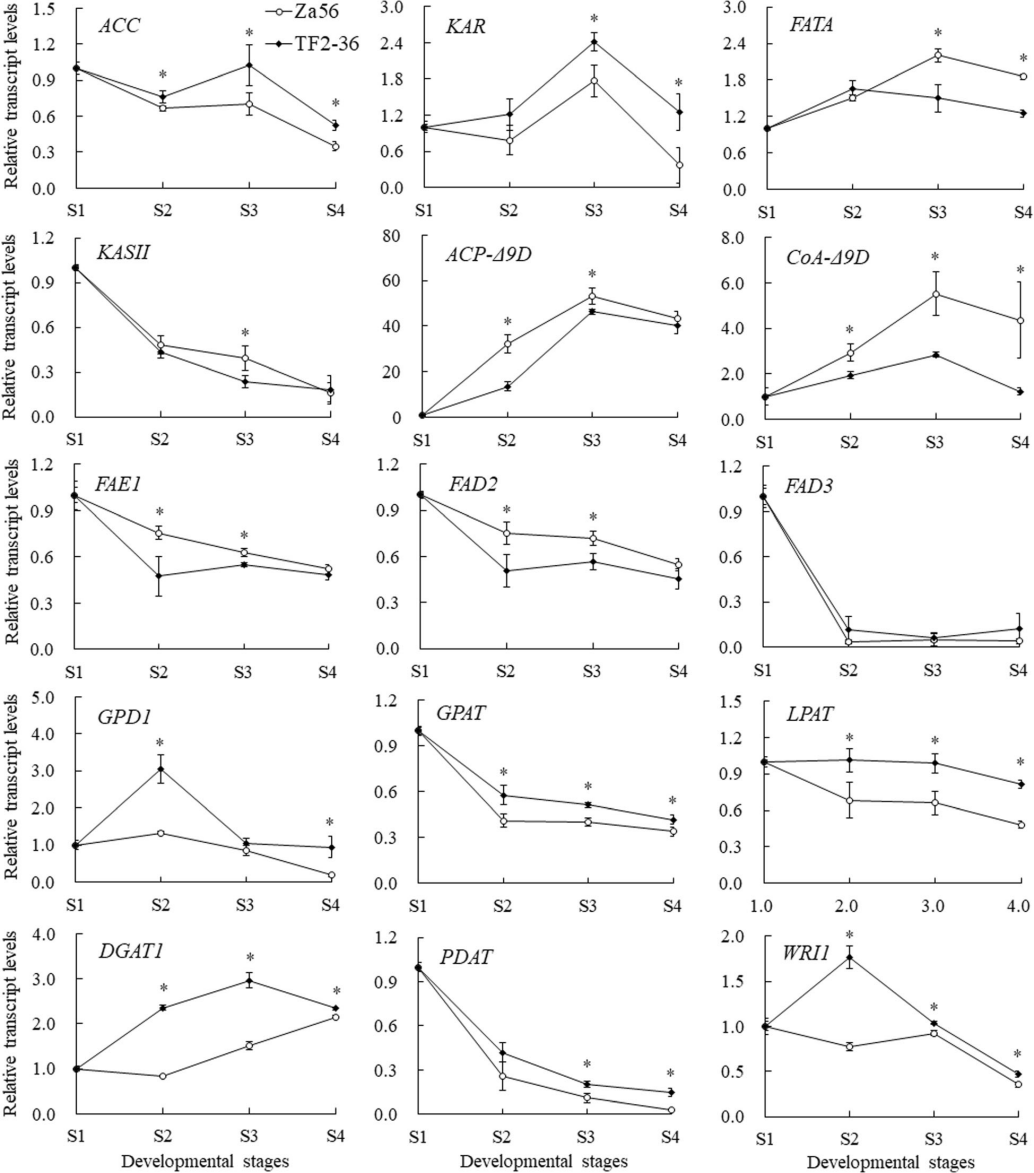
qRT-PCR analysis of genes involved in C16:1n7 biosynthesis and TAG accumulation in lines ‘Za56’ and ‘TF2–36’ at four different developmental stages

In plants, FA and oil accumulation in non-seed tissues involve several organelles and multiple enzymatic reactions, and there is no available genetic information on the regulatory mechanism of C16:1n7 and TAG biosynthesis in SBP (non-seed tissue). Based on previous studies [4, 18, 35] and the KEGG pathway analysis results (Additional file 5: Table S5), we selected 15 DEGs to determine by qRT-PCR whether the expression pattern during SBP development in lines ‘Za56’ and ‘TF2–36’ correlated with C16:1n7 biosynthesis and the changes in oil content. Nine candidate genes were involved in the FA biosynthesis pathway, namely, *ACC* (c135286_g1_i2), *KAR* (c119073_g1_i1), *FATA* (c141493_g1_i2), *KAS II* (c147281_g2_i3), *FAE1* (c108825_g1_i1), *ACP-Δ9D* (c108934_g1_i3), *CoA-Δ9D* (c119361_g2_i1), *FAD2* (c129094_g1_i1), and *FAD3* (c146563_g1_i2). Five candidate genes, namely, *GPD1* (c138230_g1_i1), *GPAT* (c131644_g1_i1), *LPAT* (c129657_g2_i2), *DGAT1* (c144982_g1_i2), and *PDAT* (c133634_g3_i1), were involved in the TAG biosynthesis pathway (Fig. 6). One TF *WRI1* (c141864_g1_i1) was associated with FA and oil biosynthesis-related genes expression and regulation. The expression levels of these 15 unigenes in S1, S2, S3, and S4 were mostly consistent between the qRT-PCR and RNA-seq experiments (Fig. 8), which indicated that the expression profiles in our transcriptomics results had a high confidence level.

*ACP-Δ9D*, *CoA-Δ9D*, *KASII*, and *FAE1* were important coordinated regulatory genes related to C16:1n7 biosynthesis (Fig. 6). The relative expression levels of *ACP-Δ9D* and *CoA-Δ9D* first increased from S1 to S3 and then decreased in the berry pulp from both lines (Fig. 8). In particular, the peak expression of these two genes in line ‘Za56’, with a high C16:1n7 content, were higher than those in line ‘TF2–36’, with a low C16:1n7 content, in S3 (Fig. 2). However, *KASII* and *FAE1* expression in both lines were significantly downregulated from S1 to S4, which corresponded to the decreasing trend in C18:0 and C18:1n7 contents (Fig. 2). The *ACC* expression in developing berry pulp from line ‘TF2–36’ was significantly higher than that in line ‘Za56’, and the peak *KAR* expression in line ‘TF2–36’ was higher than that in line ‘Za56’, with lower C16:0 contents. The expression of the *FATA* gene in line ‘Za56’ berry pulp was higher than that in line ‘TF2–36’ in S3 and S4. The downregulated expression of the *FAD2* and *FAD3* genes during berry pulp development in both lines was consistent with the decreasing contents of C18:2 and C18:3, respectively (Fig. 2).

*GPD1* first increased at S1–S2 and then declined from S3 to S4 in developing berry pulp from both lines (Fig. 8). The expression levels of *GPAT*, *LPAT*, and *PDAT* in both lines decreased from S1 to S4. In developing berry pulps of line ‘TF2–36’ *DGAT1* expression increased from S1 to S3 but increased from S2 to S4 in line ‘Za56’. *WRI1* in line ‘TF2–36’ sharply increased at S1–S2 and then declined from S2 to S4, which was similar to the expression pattern of *GPD1* in developing berry pulps of line ‘TF2–36’. The relative expression levels of *GPD1*, *GPAT*, *LPAT*, *DGAT1*, *PDAT*, and *WRI1* in developing berry pulp from line ‘TF2–36’, with a high oil content, were higher in every stages than those in line ‘Za56’, with a low oil content (Fig. 1). Interestingly, the coordinated high expression of *GPD1* and *WRI1* in the mid-early developmental stages and *DGAT1* in the mid-late stages contributed to increased biosynthesis of the TAG in SBP.

## Discussion

Palmitoleic acid, an omega-7 FA, has numerous health benefits, such as protecting the cardiovascular system and inhibiting oncogenesis [8]. Due to the high value of and growing demand for omega-7 FAs, exploiting oleaginous resources and dissecting the mechanism of C16:1n7 biosynthesis and oil accumulation have been extensively pursued [12, 36–38]. Recently, sea buckthorn has gained popularity as a resource in the food and nutraceutical industries [1] because C16:1n7 is synthesized at very low levels in common oil crop seeds but can accumulate to 0.1–12% of all FAs in sea buckthorn seed oil [39, 40] and, more notably, accounts for 12–52% of all FAs in SBP oil [3, 41], with a ratio of MUFA to SFA of approximately 1:1 [28]. SBP oil with a high C16:1n7 content is a very balanced source of MUFAs for human health and nutrition, but the molecular mechanism of high C16:1n7 accumulation is still poorly understood. Therefore, considerable research efforts related to the exploration of C16:1n7 biosynthesis and TAG accumulation in SBP (non-seed tissue) are urgently needed. To explore whether transcriptional regulation is involved in controlling C16:1n7 biosynthesis and TAG accumulation in SBP, we analysed the dynamic patterns in oil content and FA composition, especially for C16:1n7, in developing berry pulps from lines ‘Za56’ and ‘TF2–36’ (Fig. 1). Of these two lines, line ‘Za56’ has a higher C16:1n7 content than that in line ‘TF2–36’. We carried out comparative transcriptomics analysis in the two lines at four berry pulp developmental stages and generated 183,235,418 and 214,587,718 clean reads for lines ‘Za56’ and ‘TF2–36’, respectively (Table 1). Bioinformatics analysis identified a total of 323,881 unigenes, 122,305 of which were annotated, in developing SBP. A total of 139 DEGs (42 functional genes) were found to be involved in lipid metabolism. Of these, 65 were involved in FA biosynthesis and 74 were associated with TAG accumulation (Table 3), suggesting that these DEGs regulate FA biosynthesis and TAG accumulation in SBP. The coordinated expression of genes involved in FA biosynthesis and TAG accumulation (*ACC*, *KAR*, *FATA*, *KAS II*, *FAE1*, *ACP-Δ9D*, *CoA-Δ9D*, *FAD2*, *FAD3*, *GPD1*, *GPAT*, *LPAT*, *DGAT1*, *PDAT*, and *WRI1*) contributes to the high levels of C16:1n7 in SBP oil. These results provide a scientific basis for understanding the mechanisms of C16:1n7 biosynthesis and accumulation in berry pulp (non-seed tissue) and are valuable to the genetic breeding programme for achieving a high quality and yield of SBP oil.

### FA composition and oil content during berry pulp development in sea buckthorn

Because berry pulp and seed oils have unique bioactive compounds (FAs, phytosterols, carotenoids, and tocopherols), sea buckthorn berries have been used for hundreds of years in Russia and China for medicinal and nutritional purposes. Monounsaturated palmitoleic acid is rare in oil crops, such as soybean (relative C16:1n7 content = 0.14%), rapeseed (0.24%), and olive (0.73) [42], and a few rare plants, such as *Kermadecia sinuate* (70%), *Doxantha unguis-cati* (64%), and *Tasmanian waratah* (45%), are known to accumulate C16:1n7 in the seeds [8]. However, unlike these plants that accumulate C16:1n7 in the seeds, SBP oil has a C16:1n7 content of approximately 40% suitable for commercial extraction [2–4] In fact, approximately 40% of plant oils are extracted from non-seed tissues [43], and different amounts of C16:1n7 can be synthesized in different non-seed tissues, for example, 5.36% in the pericarp of bayberry [44], 0.41% in the leaves of tobacco [45], and trace amounts in the mesocarp of oil palm [17] and Chinese tallow [46]*.* Regarding C16:1n7 biosynthesis, the underlying mechanistic details are not yet well understood, particularly in non-seed tissue with high C16:1n7 accumulation.

Sea buckthorn is a unique species with a high accumulation of C16:1n7 in non-seed tissue (berry pulp) [8, 47], and the molecular mechanism of C16:1n7 biosynthesis and accumulation in berry pulp has not yet been reported. In this study, we measured the composition of the main FAs, especially C16:1n7, and the oil contents in developing berry pulp from the sea buckthorn lines ‘Za56’ (hybrid progeny of ssp. *mongolica* and ssp. *sinensis*) and ‘TF2–36’ (ssp. *mongolica*), with a closed genetic relationship [48]. The C16:1n7 content was the highest among all FAs in mature berry pulp from both line ‘Za56’ (41%), with a low oil content (4.8–8.5%), and line ‘TF2–36’ (39%), with a high oil content (9.3–35.4%) (Figs. 1 and 2). The contents of C16 FAs (C16:0 and C16:1n7) in mature pulp (S4) were significantly higher than those in immature pulp (S1) from both lines, while the contents of C18 FAs (C18:0, C18:1n9, C18:2, and C18:3) sharply decreased during berry pulp development (Fig. 2). As reported previously, the content of C16 FAs sharply decreased from 28.77 to 6.06% in developing seeds of sea buckthorn line ‘SJ1’, and C18 FAs accumulated to high levels in mature seeds (93.94%), which were much higher than those in immature seeds (71.23%) [35]. We also found similar accumulation patterns of C16 and C18 FAs between the two tissues in one line, ‘XE3’ [2]. The results of comparative analysis among these selected samples indicate that the metabolic flux from C16:0 to C16:1n7 or C18:0 plays an important role in determining the composition of FAs and TAG and provide the critical basis for understanding the biosynthesis and accumulation mechanism of berry pulp (non-seed tissue) with a high oil content.

### Coordinated regulation of multigenes contributes to C16:1n7 accumulation in SBP

The sea buckthorn transcriptome libraries for developing berry pulps were first constructed, and 65 DEGs involved in FA biosynthesis and 74 DEGs associated with TAG accumulation were revealed (Table 3). We focused on C16:1n7 biosynthesis and the associated TAG accumulation pathway (Fig. 6) to confirm the gene expression patterns of related key enzymes in developing berry pulp from sea buckthorn lines ‘Za56’ and ‘TF2–36’ (Figs. 7 and 8).

The key steps in the biosynthesis and accumulation of C16:1n7 and TAG in SBP are outlined in red in Fig. 6. Three major biosynthesis events occur during the production of C16:1n7. The first involves the biosynthesis of C16:0-ACP and/or C16:1n7-ACP in plastids and the export of these molecules to the cytosol. The second involves the formation of C16:0-CoA and/or C16:1n7-CoA and the transfer of these products to the ER, where C16:0-CoA can be further converted to C16:1n7-CoA. The third involves the incorporation of nascent C16:1n7 into TAGs, which subsequently accumulate in oil bodies. Previous studies revealed that the Δ9D enzyme catalyses the desaturation of C16:0 to form C16:1n7, and some Δ9D enzymes can also convert C18:0 to C18:1n9 [8]. Numerous *Δ9D* genes from various plants have been cloned and characterized so far [37, 49, 50], and many studies have verified the expression of *Δ9D* genes capable of converting C16:0 to C16:1n7 in transgenic plants, including *Arabidopsis*, *Nicotiana tabacum*, *Glycine max*, and *Brassica napus* [12, 36, 38, 51]. In this study, we discovered two distinct classes of Δ9D enzymes, designated ACP-Δ9D (soluble) and CoA-Δ9D (membrane-bound), in SBP. The higher levels of *ACP-Δ9D* (1–53.2) and *CoA-Δ9D* (1–5.5) expression in developing SBP from line ‘Za56’ than in line ‘TF2–36’ (1–46.4 and 1–2.8, respectively) (Fig. 8) were consistent with the higher accumulation of C16:1n7 in ‘Za56’ (13.3–41.3%) than in ‘TF2–36’ (12.6–39.0%) (Fig. 2b and Additional file 2: Table S2). Gao et al. [52] found that the ACP-Δ9D enzyme desaturated C16:0 to C16:1n7 with high activity and that the C16:1n7 content in transgenic tobacco leaves overexpressing the *Macfadyena unguis-cati ACP-Δ9D* gene significantly increased to 16.4–20.4% compared to that in wild-type leaves (< 0.4%). Cytosolic *CoA-Δ9D* can also function in plastids of plant cells; seed-specific expression of a yeast *CoA-Δ9D* in soybean seeds resulted in (10.5%) plastid C16:1 n7 production higher than that in wild-type soybean seeds (< 0.1%) [36]. Furthermore, co-expression of a castor *ACP-Δ9D* gene in plastids and two fungal (*Stagonospora nodorum* and *Aspergillus nidulans*) *CoA-Δ9D* genes in the ER can increase the C16:1n7 content from 1.9 to 43% in transgenic *Arabidopsis* seeds [51].

However, we found that the coordinated upregulation of *ACP-Δ9D* and *CoA-Δ9D* and downregulation of *KASII* and *FAE1* was critical in SBP oil biosynthesis to achieve high C16:1n7 accumulation (Figs. 6 and 8). During the development of SBP, an increasing total C16 FA (C16:0 and C16:1n7) content coupled with a sharp decline in C18 FA content; the content of C16 FAs was significantly higher than that of C18 FAs in S2–S4 (Additional file 2: Table S2). The length and desaturation of FA carbon chains are regulated by the activity of *KASII*, *FAE1*, *Δ9D, FAD2*, and *FAD3*. *KASII* competes with *ACP-Δ9D* for substrate and elongates the majority of C16:0-ACP to C18:0-ACP and/or C16:1n7-ACP to C18:1n7-ACP; *FAE1* is responsible for the extraplastidial elongation of C16:1n7-CoA to C18:1n7-CoA [8]. Therefore, *KASII* was strongly downregulated in developing SBP, resulting in an increased accumulation of C16:0-ACP, which is then desaturated by ACP-Δ9D to produce C16:1n7-ACP. Furthermore, the higher CoA-Δ9D activity coordinated with the downregulation of *FAE1* led to the increasing accumulation of C16:1n7-CoA. Previous studies revealed that high *KAS II* expression was consistent with the high proportion of C18 FAs in peony seeds [53] and that the suppression of *KAS II* by short hairpin RNAi led to an increase in C16:0 accumulation in palm seed oil [54]. Expression of castor *ACP-Δ9D* gene in wild-type *Arabidopsis* seeds increased C16:1n7 accumulation from 0.1 to 1.6%, yielding a total of 14.4% omega-7 FA, furthermore, the overexpression of *ACP-Δ9D* in a *fab1* (KASII) mutant increased the accumulation of C16:1n7 to 23.5%, and the expression of *ACP-Δ9D* in the *fab1 fae1* double mutant resulted in an increase in C16:1n7 to 26.2% in transgenic seeds [51].

The downregulation of *KASII* correlated with the decreased content of C18:0, which was only approximately 0.8% in mature berry pulp (S4). Moreover, the content of C18 unsaturated fatty acids (UFAs) (C18:1n9, C18:1n7, C18:2n6, and C18:3n6) also sharply declined (Figs. 2 and 8). Although ACP-Δ9D also catalyses the conversion of C18:0-ACP to C18:1n9-ACP [55], storage lipid synthesis is restricted by the supply of precursors [56]. Therefore, the sharp decline in C18:1n9 content in developing SBP was due to a lack of the C18:0-ACP substrate (Fig. 2). However, the decreased contents of C18:2n3 and C18:3n6 in berry pulp from both sea buckthorn lines were associated with the downregulation of the *FAD2* and *FAD3* genes, respectively (Figs. 2 and 8). Interestingly, in our previous study, we found that the high accumulation of C18 UFAs (88% of the total FAs) in sea buckthorn seed oil was due to low expression of the *CoA-Δ9D* gene coordinated with high expression of the *KAS II*, *ACP-Δ9D*, *FAD2*, and *FAD3* genes [28]. These results are significant for understanding the mechanism of high C16:1n7 accumulation in SBP (non-seed tissue) and provide a scientific basis for enhancing C16:1n7 biosynthesis in non-seed tissues by means such as overexpressing *Δ9D*, downregulating *KASII*, co-expressing *ACP-Δ9D* in plastids and *CoA-Δ9D* in the ER, and optimizing the metabolic flux toward TAG assembly.

### Source and sink genes control TAG biosynthesis and assembly in SBP

The glycerol or G3P supply is co-limiting for lipid synthesis; attempts to improve TAG synthesis solely by increasing the FA supply may not be successful and will need approaches that lead to a simultaneous increase in both FAs and G3P [19]. *GPD1* and *DGAT* are described as the ‘source’ and ‘sink’ genes, respectively, in TAG biosynthesis; regulating the expression of these two genes can increase oil content in plant tissues [57]. GPD1 is a rate-limiting enzyme in lipid synthesis [58], and G3P, a primary substrate for TAG biosynthesis, can be produced via a reaction catalysed by *GPD1* during glycerol synthesis [59]. DGAT and PDAT catalyse the conversion of DAG to TAG [20, 60]. In addition, WRI1 TF regulates the expression of genes involved in FA and TAG biosynthesis [21]. In the present study, we confirmed the differential expression levels of genes involved in TAG biosynthesis and accumulation in the developing pulp of two sea buckthorn lines, ‘Za56’ and ‘TF2–36’. The *GPD1*, *GPAT*, *LPAT*, *DGAT1*, and *PDAT* genes were more highly expressed in line ‘TF2–36’ (with a high pulp oil content) than in line ‘Za56’ (with a low pulp oil content) by qRT-PCR (Fig. 8), consistent with the data for the oil contents in the berry pulp from the two lines (Fig. 1). However, the expressions of *GPD1* and *DGAT1* genes had obviously upregulated trend, and *GPAT*, *LPAT*, and *PDAT* expressions always decreased during the development stages of both lines (Fig. 8). Therefore, we concluded that the coordinated high expression of *GPD1* and *DGAT1* genes resulted in high oil accumulation in SBP.

The peak *GPD1* expression in lines ‘Za56’ and ‘TF2–36’ appeared during the period of rapid oil biosynthesis (mid-early stages), and the highest oil contents in the pulp were 8.48 and 35.42%, respectively, in S3 (Fig. 1), which was after the stage of peak *GPD1* expression (S2) (Fig. 8). Therefore, the high expression of the *GPD1* gene contributed to increased synthesis of the TAG precursor G3P in the early stages of berry pulp development. *In planta* feeding, glycerol leads to increased levels of G3P and TAG in developing *Brassica napus* seeds, and the oil content peak lagged behind that of G3P for 15 d [19]. In addition, Vigeolas et al. [61] found that a twofold overexpression of the yeast gene coding for GPD1 led to a three- to fourfold increase in the level of G3P in transgenic *Brassica napus* seeds, resulting in a 40% increase in oil content, with the protein content remaining substantially unchanged. Overexpression of maize *GPD1* remarkably enhanced the G3P production and the salinity/osmotic stress tolerance in transgenic *Arabidopsis* plants, and the transgenic lines showed no aberrant phenotype under normal growth conditions, no matter in the vegetative growth phase or reproductive developmental stages [62]. Overexpression of the yeast *GPD1* increased *Camelina sativa* seed oil content about 5.4% but cause no change in seed protein content and no effect on seed germination and seedling growth [63].

The peak *DGAT1* expression in berry pulp from line ‘TF2–36’ (with a high oil content) appeared during the period of stable oil accumulation (mid-late stages) and was significantly higher than that in line ‘Za56’ (with a low oil content) in S3. Therefore, the high expression of *DGAT1* catalysed the robust assembly of acyl-CoA FAs and DAG to form TAG in the late stages of berry pulp development. Li et al. [24] found that *DGAT1* appears to be a major enzyme for seed oil accumulation in *Arabidopsis* and soybeans. Overexpression of DGAT cDNA in wild-type *Arabidopsis* seeds increased the oil content by 11–28% [64]. In addition, seed-specific overexpression of *AtDGAT1* in *Brassica juncea* seed improved the oil content significantly, with a maximum oil content increase of 8.3% in the transgenic plants compared to that in the wild-type plants [65]. In addition, we examined the expression profiles of the *GPD1* and *DGAT1* genes in seeds and berry pulp tissues of one sea buckthorn line, ‘XE3’, and found that these two genes had higher expression in pulp (with an oil content of 36.90%) than in seeds (14.15%) [66].

Metabolic engineering strategies for increasing plant lipids can be described as the surprising synergy when ‘Push’ approach (up-regulation of substrate biosynthesis by *WRI1*) and a ‘Pull’ approach (increasing TAG assembly by *DGAT*) are combined [67]. This strategy corresponded to the coordinated high expression of source gene (*GPD1*) and sink gene (*DGAT*) contributes to increase oil content in plant tissues [57]. In developing SBP, we found similar expression patterns between *WRI1* and *GPD1*, and their expressions of line ‘TF2–36’ were higher than those in line ‘Za56’ with a sharp increase in the mid-early stages (Fig. 8). The coordinated high expressions of *WRI1* and *GPD1* in line ‘TF2–36’, with a high oil content, suggested to play a major role in the high levels of storage oils in SBP. Overexpression of the *WRI1* in *Arabidopsis* and maize yielded higher TAG levels in seed [68, 69] while an *Arabidopsis wri1* knockout mutant exhibit reduced seed oil content [70]. Vanhercke et al. [67] found that overexpression of the *Arabidopsis WRI1* gene led to a 22-fold increase TAG levels in transgenic *Nicotiana benthamiana* dry leave, and the C16:1 content increased from 0.4 to 0.9%. Furthermore, when both the *Arabidopsis WRI1* and *DGAT1* genes were co-infiltrated (push and pull approach), a significant synergistic effect on leaf oil accumulation was observed with TAG levels increasing almost 100-fold and making up 2.48% of the leaf dry weight. In addition, *Camelina sativa* plants co-expressing a yeast cytosolic *GPD1* and the *Arabidopsis DGAT1* genes exhibited up to 13% higher seed oil content compared to wild-type plants, and these coexpressing lines showed significantly higher seed oil yields than the plants expressing *GPD1* and *DGAT1* alone [63]. Therefore, combining the overexpression of lipid biosynthetic genes, *WRI1*, *GPD1*, and *DGAT1*, appears to be a positive strategy to achieve a synergistic effect on the flux through the TAG biosynthesis pathway, and thereby further increase the oil yield. This finding suggests that the coordinated high expression of *WRI1* and *GPD1* in the mid-early stages and *DGAT1* genes in the mid-late stages resulted in TAG assembly and oil accumulation in SBP.

## Conclusion

Palmitoleic acid (an omega-7 FA) is rarely synthesized in common oil crops [8] but is an essential unusual FA that has a wide range of biological activities and benefits to human health [9–11]. SBP is a good source of bioactive edible oil (2–38%) with a high level of C16:1n7 (approximately 40%), and the global demand for sea buckthorn pulp oil is increasing [1–4]. However, the regulatory mechanism of C16:1n7 biosynthesis and TAG accumulation in SBP is poorly understood. In this study, we first generated a comprehensive transcriptome of berry pulp from two lines, ‘Za56’ and ‘TF2–36’, in four developmental stages. Oils and C16:1n7 predominantly accumulated in mature berry pulp stages, and the C16:1n7 contents in developing berry pulp from line ‘Za56’ (with a low oil content) were higher than those in line ‘TF2–36’ (with a high oil content). We identified 139 DEGs involved in lipid metabolism between the different developmental stages of berry pulp in the two lines. Furthermore, expression analysis of some key genes involved in FA biosynthesis and TAG assembly revealed that high expression of the *ACP-Δ9D* and *CoA-Δ9D* genes coordinated with downregulation of the *KASII* and *FAE1* genes is a critical event for high C16:1n7 accumulation and that the coordinated high expression of *GPD1*, *WRI1*, and *DGAT1* accelerated TAG accumulation in SBP. These results provide the first scientific basis for understanding the mechanism of C16:1n7 biosynthesis and accumulation in SBP (non-seed tissue) and are valuable to the genetic breeding programme for achieving a high quality and yield of SBP oil with a high level of C16:1n7.

## Methods

### Plant materials

Samples were collected from cultivated *Hippophae* L. trees (lines ‘Za56’ and ‘TF2–36’) growing in Institute of Berries at Heilongjiang Academy of Agricultural Sciences (voucher No. 02901, identified by Jin-you Shan and deposited at HAAS), Suiling county, Heilongjiang, China (47°14′12.3“ north latitude, 127°05’39.9” east longitude) under natural climate conditions. The orchard had a mean annual rainfall of 570.6 mm, a mean annual temperature of 2.0 °C, a mean annual evaporation capacity of 1242.5 mm and an effective accumulative temperature of 2460.4 °C [71]. The collection of all samples completely complies with local and national legislation permission. Line ‘Za56’ was selected from seedlings of the hybrid progeny of ssp. *mongolica* (♀) and ssp. *sinensis* (♂), and line ‘TF2–36′ was selected from seedlings of ssp. *mongolica*. The molecular marker-based genetic similarity of these two lines is 0.752 [48]. The three trees of each line selected as biological triplicates for fruit collection were cutting seedlings. The developing fruits of lines ‘Za56’ and ‘TF2–36′ were collected on July 6 (S1), July 28 (S2), August 19 (S3) and September 10 (S4), 2015. These samples were immediately frozen in liquid nitrogen and stored at − 80 °C until use. Eight RNA sequencing (RNA-seq) transcriptome libraries were constructed based on the developmental berry pulp from these two lines.

## Additional files

Additional file 1: **Table S1.** Primers used for qRT-PCR of multigenes involved in FA biosynthesis and TAG accumulation in sea buckthorn berry pulp. (XLSX 14 kb)

Additional file 2: **Table S2.** FA composition in sea buckthorn berry pulp from lines ‘Za56’ and ‘TF2–36’ at four developmental stages. (XLSX 13 kb)

Additional file 3: **Table S3.** Functional classifications of unigenes in developing berry pulps of sea buckthorn based on COG category and KEGG pathway. (XLSX 12 kb)

Additional file 4: **Table S4.** Differentially expressed genes for each of the 16 pairwise comparison groups. (XLSX 3263 kb)

Additional file 5: **Table S5.** Unigenes involved in FA biosynthesis and TAG accumulation based on KEGG pathway analysis. (XLSX 44 kb)

## Abbreviations

ACC: Acetyl-CoA carboxylase
ACP: Acyl carrier protein
ACP-Δ9D: Delta9-ACP desaturase
CoA-Δ9D: Delta9-CoA desaturase
DEG: Differentially expressed gene
DGAT1: Diacylglycerol O-acyltransferase 1
ER: Endoplasmic reticulum
FA: Fatty acid
FAD2: Fatty acid desaturase 2
FAD3: Fatty acid desaturase 3
FAE1: Fatty acid elongation 1
FAME: Fatty acid methyl ester
FATA: Fatty acyl-ACP thioesterase A
FPKM: Fragments per kilobase of exon model per million mapped fragments
G3P: Glyceraldehyde 3-phosphate
GC-TOF/MS: Gas chromatography time-of-flight mass spectrometry
GPAT: Glycerol-3-phosphate acyltransferase
GPD1: Glycerol-3-phosphate dehydrogenase
KAR: 3-ketoacyl-ACP reductase
KAS II: 3-ketoacyl-ACP synthase II
LPAT: Lysophosphatidic acid acyltransferase
MUFA: Monounsaturated fatty acid
PDAT: phospholipid diacylglycerol acyltransferase
PUFA: Polyunsaturated fatty acid
SFA: Saturated fatty acid
TAG: Triacylglycerol
TIC: Total ion chromatography
UBQ5: Ubiquitin 5
WRI1: WRINKLED1
